# Classification of Brain MRI Tumor Images Based on Deep Learning PGGAN Augmentation

**DOI:** 10.3390/diagnostics11122343

**Published:** 2021-12-13

**Authors:** Ahmed M. Gab Allah, Amany M. Sarhan, Nada M. Elshennawy

**Affiliations:** 1 Department of Computers and Control Engineering, Faculty of Engineering, Tanta University, Tanta 31733, Egypt; amany_sarhan@f-eng.tanta.edu.eg (A.M.S.); nada_elshennawy@f-eng.tanta.edu.eg (N.M.E.); 2 Department of Information Systems, Faculty of Computers and Artificial Intelligence, University of Sadat City, Sadat City 32897, Egypt

**Keywords:** brain tumor, magnetic resonance imaging, deep learning, generative adversarial network, convolutional neural network

## Abstract

The wide prevalence of brain tumors in all age groups necessitates having the ability to make an early and accurate identification of the tumor type and thus select the most appropriate treatment plans. The application of convolutional neural networks (CNNs) has helped radiologists to more accurately classify the type of brain tumor from magnetic resonance images (MRIs). The learning of CNN suffers from overfitting if a suboptimal number of MRIs are introduced to the system. Recognized as the current best solution to this problem, the augmentation method allows for the optimization of the learning stage and thus maximizes the overall efficiency. The main objective of this study is to examine the efficacy of a new approach to the classification of brain tumor MRIs through the use of a VGG19 features extractor coupled with one of three types of classifiers. A progressive growing generative adversarial network (PGGAN) augmentation model is used to produce ‘realistic’ MRIs of brain tumors and help overcome the shortage of images needed for deep learning. Results indicated the ability of our framework to classify gliomas, meningiomas, and pituitary tumors more accurately than in previous studies with an accuracy of 98.54%. Other performance metrics were also examined.

## 1. Introduction

Cancer is considered one of the most widespread causes of mortality around the world. Cancers are complex diseases that may afflict every part of the human body. Poor classification of tumors may be attributable owing to the wide variation in severity of the disease, duration of illness, location of tumors, and degrees of sensitivity or resistance to the various chemotherapeutic drugs [1]. There has been a significant rise in the number of patients with brain tumors over the past decade, with cancers of the brain becoming the 10th most common type of tumor in children and adults alike. Accurate classification of brain tumors would clearly result in the most effective medical intervention and can greatly increase survival among patients.

Computer-aided diagnosis (CAD) techniques have been helping neuro-oncologists in multiple respects. CAD techniques have aided in the early detection and classification of brain tumors [2]. Physicians aided by CAD are able to make more accurate classifications compared with those relying on visual comparisons alone [3]. MRIs contain valuable information regarding the type, size, shape, and position of brain tumors without subjecting the patient to harmful ionizing radiation [4]. MRIs provide higher contrast of soft tissues compared with computerized tomography (CT) scans. Thus, coupled with a CAD system, MRIs can quickly help identify the location and size of tumors [5,6].

Advances in computer have yielded powerful tools that help in obtaining more accurate diagnoses. One such advance was the creation of deep learning-based systems, whose introduction has resulted in a noticeable improvement in medical image analysis and treatment-related decision making [7], especially when deep neural networks-based technology is used by trained specialists [8]. With the rapid development of deep learning techniques and their ability to better classify medical images, CAD has become a more widely applied method of diagnosis among medical imaging specialists [9]. Expanding research in applying deep learning to the classification of various diseases, within the limits of available technology, is currently a top priority of radiology researchers.

Of the multitude of deep machine learning (ML) techniques, CNNs have been extensively used in the medical image analysis of different diseases and therefore widely used by the research community. CNNs yield more accurate image-based classification and prediction of prognosis of such tumors/diseases as lung cancer [10,11], pneumonia [12], colon cancer [13], and liver disease [14]. CNNs have also been used to create a deep learning algorithm for human skin detection as a part of dermatology diagnostics [15], Moreover, CNNs were incorporated in a model of brain tumor detection and segmentation [16] and more recently in a model of COVID-19 classification [17] without human involvement.

While CNNs successfully aid in single-label image classification, most real-world images generally contain multiple labels with different objects, proprieties, and attributes within a single image. Unlike other forward neural networks, the recurrent neural network (RNN) is a neural network that utilizes multi-label image classification to learn independent classifiers for each image attribute. RNNs also capture dynamic information in serialized data by hiding the periodic connections of nodes in layers and are thus able to classify serialized data [18]. There are different types of RNNs such as Bidirectional-LSTM (Bi-LSTM) and Bidirectional-GRU (Bi-GRU). As shown by a number of reported studies, the combination of CNN and RNN (the Gated Recurrent Unit and Long Short-Term memory networks model) results in models with high disease-classification accuracy [19,20].

One of the obstacles to reaching the optimal performance of a deep learning model is the lack of a sufficient sample size in a data set. Therefore, data augmentation has been used in several recent research studies in an attempt to enlarge data sets. This allows CNNs to explore the invariances in a data set, leading to robust training models. Data augmentation helps solve the problem of overfitting in CNNs by artificially enlarging the data set in both diversity and number using a number of transformations, including rotations, flips, as well as random transformations [21,22,23].

A generative adversarial network (GAN) was introduced in 2014 by I. Goodfellow et al. [24]. It is a deep machine learning model that can synthesize a new image from a latent vector. It consists of two primary models: a generator and a discriminator. The generator model trains to generate new images by mapping the latent space to points in the image space. The discriminator model trains to classify synthesized images as either real or fake. The two models are trained together to generate synthesized images with highly realistic data (e.g., faces, buildings, rooms, etc.) that can fool humans.

As tool of data augmentation, the GAN model has significantly aided in solving the problem of deep learning overfitting. For example, in the GAN model proposed in [25], the Deep Convolutional Generative Adversarial Network (DCGAN), both generator and discriminator are implemented using a CNN network but are unable to reach the proposed resolution of 512 × 512. DCGAN can synthesize chest X-ray images and increase the labeled data set, improving classification performance by combining real and synthesized images with the training of Deep Convolutional Neural Networks (DCNNs). This allows for the detection of pathology across five classes of chest X-rays [26]. Moreover, because of the ability of this model to synthesize ‘real’ images, this approach has already been exploited successfully to conduct unsupervised training of radiologists, with DCGAN made to generate realistic samples for educational purposes [8]. Modified GANs were also used to improve the image quality for medical image application [27].

Brain tumors, according to World Health organization (WHO), can be classified into four grades (grade I to grade IV): grades I and II are classified as low-grade tumors, while grades III and IV are termed as high-grade tumors. Metastatic brain tumor treatment requires crucial medication parameters of non-chemotherapeutics, chemotherapeutics, radiation, and surgical techniques [28]. Brain tumor classification has been one of the most promising research areas of deep learning for medical purposes and is the primary basis of deciding on the primary form of treatment and the treatment process as well as predicting the possible success rate of treatment, and mapping out follow-up of the disease [29]. Some of the approaches used to perform feature selection and classification are explained in Table 1.

This work is focused on brain tumor classification with the following contributions: Three different deep learning models with the high ability to classify multiple brain tumors (glioma, meningioma, and pituitary tumors) using MRIs are discussed. They are VGG19 + CNN, VGG19 + Gated Recurrent Unit (GRU), and VGG19 + Bi-Directional Gated Recurrent unit (Bi-GRU). Two methods of data augmentation, namely, classic data augmentation and PGGAN data augmentation, are utilized to increase the data set size. A complete comparison of the models and their applications is presented. The study also discusses the shared data set introduced by Cheng et al. [30].

## 2. Materials and Methods

Gliomas, meningiomas, and pituitary tumors are three types of fatal brain tumors detectable in MRIs. If undetected in their early stage, prognoses of these types of tumors can be dire [4]. Along with meningiomas, gliomas are recognized as one of the most common primary brain tumors [40]. Pituitary adenomas are the most frequent intracranial tumors. They are associated with high rates of morbidity and mortality [41]. Each tumor type is classified by a distinct classification scheme and is of different degrees, sizes, and shapes. Based on these differences, tumor types are identified [42]. MRIs provide a superior method of classification of brain tumors. Accuracy in classifying the tumor type is essential to allow physicians to decide on the best type of treatment and the treatment plan, accurately predict prognosis, and design follow-up plans.

This section provides a concise and precise description of the experimental results, their interpretation, as well as the experimental conclusions that can be drawn.

**Figure 1:**
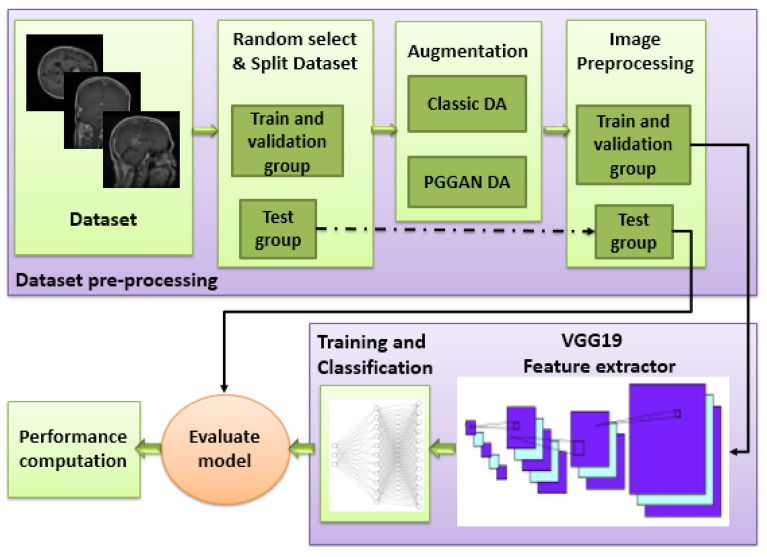
Block diagram of the proposed framework (DA: Data augmentation).

A deep learning framework was developed for the purposes of this study. As shown in Figure 1, the framework consisted of three main stages: a data set pre-processing stage; a deep learning model for feature extraction, and a classification stage. Three different models were used to classify brain tumors: VGG19 + CNN, VGG19 + GRU, and VGG19 + Bi-GRU. By accurately classifying brain tumors, this framework will reduce a radiologist’s workload and allow for more timely decision making.

### 2.1. Data set for the Study

**Figure 2:**
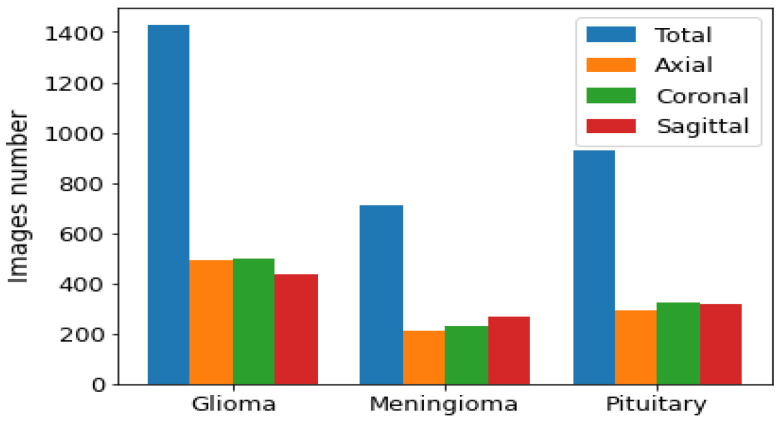
Distribution of each brain tumor type by plane.

**Figure 3:**
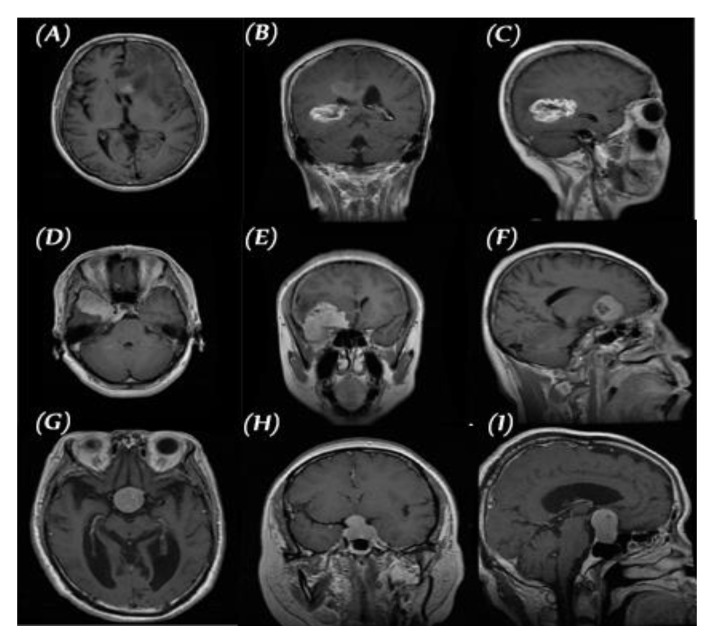
Three planes for three samples of brain tumor MR images from the primary data set, (**A**–**C**) glioma, (**D**–**F**) meningioma, and (**G**–**I**) pituitary.

We used the public data set introduced by Cheng et al. [30] in this work. This data set was created in order to synthesize images for the training of deep learning frameworks and the evaluation of how accurately they identify brain tumors on MRIs. As seen in Figure 2, the data set consisted of 3064 T1-CE MR images from 233 patients who suffered one of three different brain tumor types: gliomas (1426 images), meningiomas (708 images), and pituitary tumors (930 images). The data set’s MR images covered three planes (axial, sagittal, and coronal) with an image size of 512 × 512 pixels. A sample of images of the three tumor types from three planes is shown in Figure 3.

### 2.2. Pre-Processing and Image Augmentation

Even the smallest CNNs have thousands of parameters upon which their layers need to be trained. When using a small number of images, CNN models face the problem of overfitting. To counter this problem, we employed two methods of data augmentation (DA): classic augmentation and PGGAN-based augmentation.

The best results are typically obtained when no preprocessing is used with the MR images. Therefore, image intensities were scaled to the range [−1, 1]. The data set was randomly divided into three groups with their target label: a training group (70%), a validation group (15%), and a testing group (15%).

#### 2.2.1. Classic Data Augmentation

In this phase, the data set size was increased using an augmenter. This was achieved by changing the brain’s position enough to avoid a model memorizing the location of the brain. Classic DA applied to MRIs typically includes the following operations:

- Rotation: Rotation of image without cropping because a cropped image may not contain the whole tumor. Images were rotated at 90, 180, and 270 degrees;
- Mirroring: Images are right/left mirrored;
- Flipping: Images are up–down flipped.

**Figure 4:**
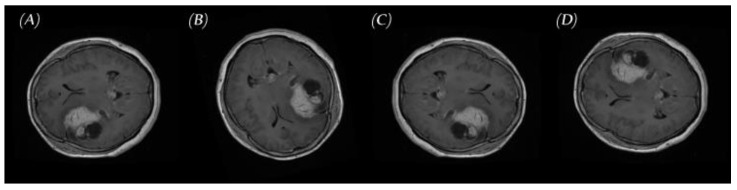
Samples of brain tumor MRIs and results of augmentation: (**A**) primary MRI, (**B**) rotation, (**C**) left-right mirroring, and (**D**) up–down flipping.

This method increased the size of the training and validation groups of the MR images in all positions: axial, sagittal, and coronal, as shown in Figure 4.

#### 2.2.2. PGGAN-Based Data Augmentation

This research investigated the possibility of using the GAN technique as another method of augmentation in order to correct the imbalance in medical data sets. PGGAN [43] is a GAN model that progressively grows off a generator and discriminator. It chooses training stability, synthesizing images up to 1024 × 1024 pixels in resolution.

**Figure 5:**
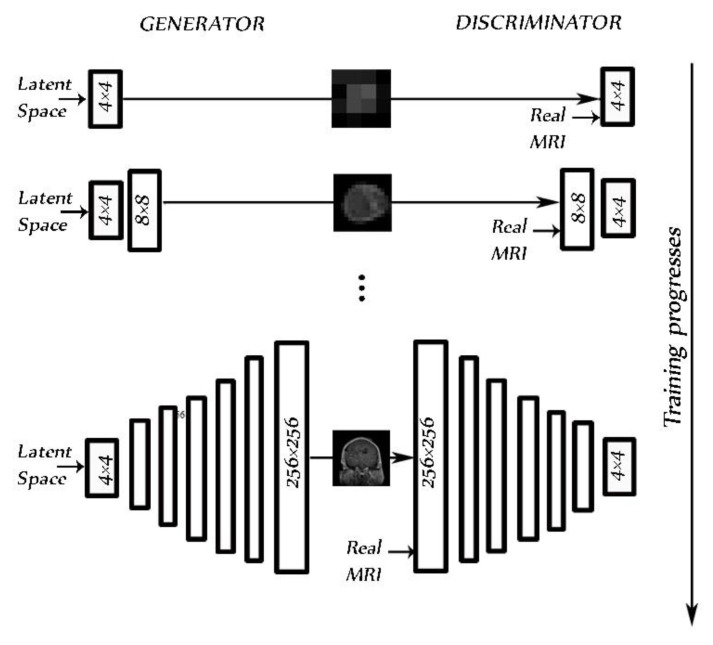
PGGAN architecture for the 256 × 256 pixel MR image brain tumor generator during training progress.

We used PGGAN to overcome the lack of sufficient images in the data set, synthesizing brain tumor MRIs in three planes: axial, sagittal, and coronal for three tumor types that had the same structure. Images were synthesized from low 4 × 4 pixel images using a 512 latent vector. Images reached a resolution of 256 × 256 pixels, increasing in resolution at each step by the power of 2 (Figure 5). We trained the model and generated each dimension in each class image separately using the main images from the data set [30].

PGGAN implementation details: The original PGGAN introduced in [43] was used. Using this PGGAN, we trained the model to synthetize MRIs of brain tumors. Ideally, a high-resolution MRI is facilitated from a latent vector of 512. The architecture of PGGAN used to synthesize the brain tumor consisted of two models: the generator and the discriminator. Variable batch size was changed during training for each resolution as follows: 4→128, 8→128, 16→128, 32→ 64, 64→32, 128→16, and 256→4, to prevent the system from exceeding available memory. An Adam optimizer [44] with a learning rate = 0.001, β1 = 0, and β2 = 0.99, and epsilon = 1 × 10^−8^ was chosen as it gives the best accuracy. We used a WGAN-GP as a loss function [45]. As in the study by [21], pixelwise feature vector normalization was applied to the generator model after each convolution layer, with the exception of the final output layer. The activation layer was a Leaky ReLU (LReLU) with a leakiness of 0.2. It was used as both generator and discriminator.

### 2.3. Proposed Deep Learning Models

Different tumor types may afflict the human brain, including, as aforementioned, gliomas, meningiomas, and pituitary tumors. MRIs represent the best radiological method of identifying and classifying these brain tumors. The application of deep machine learning has advanced the classification process of MRIs of brain tumors. The objective of this study was to develop an intelligent system that could better classify the MRIs of gliomas, meningiomas, and pituitary tumors into these three classes of tumors compared with currently existing models. To achieve this aim, first, a VGG19 was used as feature extraction.

VGG19 [46] is a widely used CNN architecture model composed of 19 layers with 3 × 3 convolution filters and a stride of 1 designed to achieve high accuracy in large-scale image applications. As a large-scale image features extractor with high accuracy, a VGG19 was essential to our framework. Several deep learning classifiers were integrated with the VGG19 extractor in order to reach maximum accuracy of brain tumor classification.

To decide on the best combination of VGG19 and classifier (VGG19 + CNN or a combination of VGG19 and an RNN model, such as GRU and Bi-GRU), we tested three different architectures: VGG19 + CNN, VGG19 + GRU, and VGG19 + Bi-GRU. We compared their performances and chose the best of them. Finally, we tested the same three models coupled with one of two types of augmentation models: a classical augmenter and a PGGAN-based augmenter (augmenters are designed to produce more realistic MRIs).

Pre-processing: Input image size stood at 224 × 224 pixels. We resized all MRIs in the data set to the same size. All PGGAN-generated images were 256 × 256 pixels in size. We thus resized those images to 224 × 224 pixels.

Data augmentation (DA) setup: To test the performance of our models, we used the following two DA setups with a sufficient number of images for the three classes of tumor:

- Classic DA;
- Classic DA + PGGAN-based DA.

#### 2.3.1. VGG19 and CNN Deep Learning Model

A deep learning model that consisted of a VGG19 + CNN model followed by CNN was designed to classify brain tumors on MRIs. It employed feature extraction and classification. In this model, VGG19 + CNN was used as a feature extraction model. The whole number of parameters of VGG19 + CNN stood at 139,581,379, which were all trainable parameters. The structure of the model is shown in Table 2; the input layer for the model was 224 × 224 × 1 brain tumor MRIs. The feature extraction consisted of a VGG19 followed by two blocks, each block having a convolution layer with 4096 neurons followed by a dropout layer with the rate of 0.5, a ReLU activation function, and finally, a SoftMax layer that functioned to classify the output into one of the three tumor types.

#### 2.3.2. VGG19 and GRU Deep Learning Model

GRU is an RNN architecture with a number of advantageous features, such as its simplicity and need for less training time, in addition to its ability to store information irrelevant to the predictions made for extended periods of time. Our second deep model, which consisted of a VGG19 model followed by GRU, was designed to classify the MRIs of brain tumors. It employed feature extraction and classification. To our second model, we added GRU as a classifier after VGG19 having been used as feature extraction. Beyond the layers of the VGG19, the output passed to a reshape layer followed by a GRU layer with 512 units, then two blocks consisting of a convolution layer of 1024 neurons followed by a dropout layer, and finally a SoftMax layer, as shown in Table 3 (27,895,747 total parameters).

#### 2.3.3. VGG19 and GRU Bidirectional Deep Learning Model

Our third model consisted of a VGG19 followed by a Bi-GRU, which was used as a feature extraction model and classifier. The model consisted of a VGG19 followed by a reshaping layer, a Bi-GRU layer, a dropout layer, a dense layer, a dropout layer, and a dense layer with a SoftMax activation function. The structure of the model is shown in Table 4 (34,714,563 total parameters).

**Figure 6:**
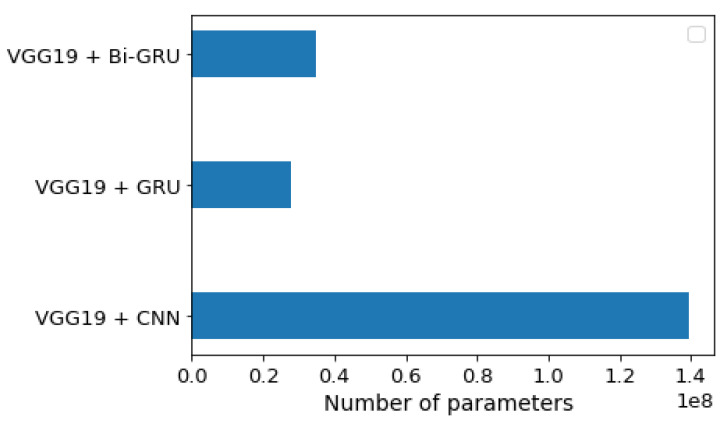
Number of trainable parameter-wise distributions for each of the three models.

Figure 6 compares the number of parameters for the three models, and the VGG19 + GRU model was shown to give a lower number of parameters than the other two models. This indicated that it had the lowest complexity. The VGG19 + CNN had a high number of parameters.

## 3. Results

All classification models in the framework created for this study were run using TensorFlow and Keras frameworks and trained using Google Colab with the following specification: 2 TB storage, 12 GB RAM, and at a minimum graphical processing of unit (GPU) P100. Before completing the final model evaluation, we conducted a number of tests to select the best hyperparameters.

We ran our model for 100 epochs with a batch size of 32 and categorical cross-entropy loss for multi-classification without ImageNet pre-training. To select the best optimizer from a choice of Adam [44], Adamax [44], RMSprop [47], or Nadam [48] for the three models, we tested the accuracy of the models using these four optimizers. We used a constant learning rate of 0.00001.

### 3.1. Performance Metrics

A number of recent studies used confusion matrices to analyze models and assess the level of performance of the classification process, categorizing relationships between data and distributions. By using different confusion matrices, classification models may be assessed extensively [49]. In our study, four primary keys—a true positive (Tp), a true negative (Tn), a false positive (Fp), and a false negative value (Fn)—were used to test the classifier. Then, based on the four outcomes, the performance of the model was computed in terms of accuracy (ACC), sensitivity, specificity (SPC), precision (PPV), negative predictive values (NPVs), the F1-score, and Matthew’s correlation coefficient (MCC).

Accuracy, as given in Equation (1), is defined as the number of correctly predicted samples to the total predicted sample.

(1) $$Accuracy\;\left(\mathrm{ACC}\right)=\frac{\mathrm{Tp}\;+\mathrm{Tn}\;}{\mathrm{Tp}\;+\mathrm{Tn}+\mathrm{Fp}+\mathrm{Fn}\;}.$$

Sensitivity (Recall), as given in Equation (2), is the number of samples labeled as positive out of the total number of positive samples.

(2) $$Sensitivity\;\left(\mathrm{Recall}\right)=\frac{\mathrm{Tp}\;}{\mathrm{Tp}\;+\mathrm{Fn}}.$$

Specificity of the true negative rate, as given in Equation (3), is the number of samples predicted as negative out of the total number of negative samples.

(3) $$Specificity=\frac{\mathrm{Tn}\;}{\mathrm{Tn}\;+\mathrm{Fp}}.$$

Precision, as in Equation (4), represents the number of samples truly positive that were predicted as such out of the total number of samples predicted as positive.

(4) $$Precision=\frac{\mathrm{Tp}\;}{\mathrm{Tp}+\mathrm{Fp}}.$$

Conversely, the negative predictive value (NPV) is the number of truly negative samples that were predicted as such, out of the total number of samples predicted as negative, given in Equation (5).

(5) $$NPV=\frac{\mathrm{Tn}\;}{\mathrm{Tn}+\mathrm{Fn}}.$$

The harmonic means of precision and recall, known as the F1-score, is shown in Equation (6).

(6) $$\mathrm{F1-score}=\frac{2*\mathrm{Tp}\;}{2*\mathrm{Tp}+\mathrm{Fp}+\mathrm{Fn}}.$$

Finally, Matthew’s correlation coefficient range allows one to gauge how well the classification model performs.

(7) $$MCC=\frac{\left(\mathrm{Tp}*\mathrm{Tn}\right)-\left(\mathrm{Fp}*\mathrm{Fn}\right)\;}{\sqrt{\left(\mathrm{Tp}+\mathrm{Fp}\right)\left(\mathrm{Tp}+\mathrm{Fn}\right)\left(\mathrm{Tn}+\mathrm{Fp}\right)\left(\mathrm{Tn}+\mathrm{Fn}\right)}}$$

In a number of previous studies, researchers chose two methods to design a classification model for brain tumors. The first method only involved axial images [31,33,38] while the second method involved images in three planes (axial, sagittal, and coronal) [4,23,30,32,34,35,36,37,39]. In our study, we used the second method to generalize the model.

### 3.2. Scenario I: Deep Learning Models with Classic Augmentation

To set the generalized design for the classification of brain tumors in all three planes, we needed to increase the total number of images in the data set. This was achieved by using a classical augmenter for all three planes. For the VGG19 + CNN model, as seen in Table 5, the highest accuracy of 96.59% was achieved using the Nadam optimizer. The other performance metrics achieved by the VGG19 + CNN model for the three classes of brain tumors are shown in Table 6. The highest performance metric involved pituitary tumors. The model achieved the highest sensitivity in identifying gliomas, for which NPV values were also the highest.

**Figure 7:**
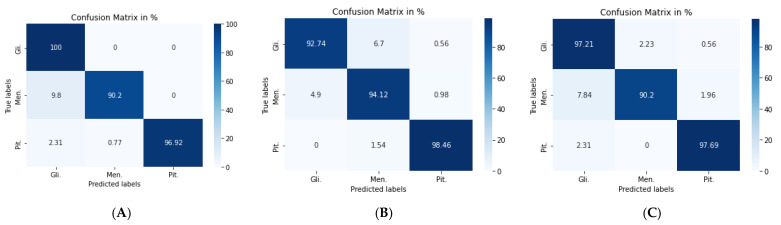
Confusion matrix for: (**A**) VGG19 + CNN model, (**B**) VGG19 + GRU model, and (**C**) VGG19 + Bi-GRU model.

Figure 7a shows the confusion matrix of the VGG19 + CNN model, in which our framework accurately identified gliomas in 100% of cases, meningiomas in 90.2% cases, and pituitary tumors in 96.92% of cases. We used a combination of CNN and RNN models to classify brain tumors in MRIs. We combined the VGG19 + GRU model; this combination enabled our model to process the characteristics of the information contained in the image and effectively learn the structural features of brain tumors.

The second model (VGG19 + GRU) combined the VGG19 as a feature extractor with GRU as a classifier. The highest accuracy of 94.89% was achieved with the Adam optimizer, as seen in Table 5, with the highest accuracy metric remaining that of pituitary tumors. Figure 7b shows the VGG19 + GRU model confusion matrix, indicating that the model successfully and accurately predicated gliomas, meningiomas, and pituitary tumors at rates of 92.74, 94.12, and 98.46%, respectively.

**Figure 8:**
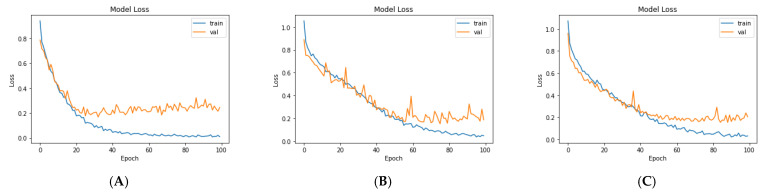
Loss over the training process for: (**A**) VGG19, (**B**) VGG19 + GRU, and (**C**) VGG19 + Bi-GRU.

For the third model (VGG19 + Bi-GRU), we used Bidirectional-GRU combined with VGG19 as a classifier of brain tumors. Table 5 shows that the VGG19 + Bi-GRU combination yielded promising results, reaching an accuracy level of 95.62% when coupled with the RMSprop optimizer. The best metrics for accuracy, precision, F1-score, specificity, and MCC were achieved in relation to the identification of pituitary tumors, while sensitivity and NPV were highest in relation to the detection of gliomas. The VGG19 + Bi-GRU model’s confusion matrix indicated successful prediction rates of 97.21, 90.20, and 97.69% of gliomas, meningiomas, and pituitary tumors, respectively. Figure 7c shows the VGG19 + Bi-GRU model confusion matrix. The losses of the three models during the entire training for the test and validation process versus epochs for the three best models is shown in Figure 8.

Even though our models achieved a high level of performance at 96.59%, they initially possessed a number of shortcomings that would have affected their application clinically. For example, training the models for the classification of a brain tumor was conducted using a limited number of images with the same characteristics and ‘increased’ in number using a classic augmenter. Therefore, we used PGGAN as an augmenter in order to improve the performance metrics of our models and make our models more realistic and generalized. Re-runs of the three models with synthesized MRIs were conducted and results compared to those of previous studies.

### 3.3. Scenario II: Deep Learning Models with PGGAN-Based DA

Increasing the accuracy of a model’s ability to classify brain tumors will naturally make our framework more reliable and clinically applicable. Therefore, the second phase of the experiment, as aforementioned, was to make synthetic MR images of brain tumors by employing the PGGAN model in order to make classification more realistic. We used nine PGGAN models: three PGGAN models for each tumor type, one model for each plane.

**Figure 9:**
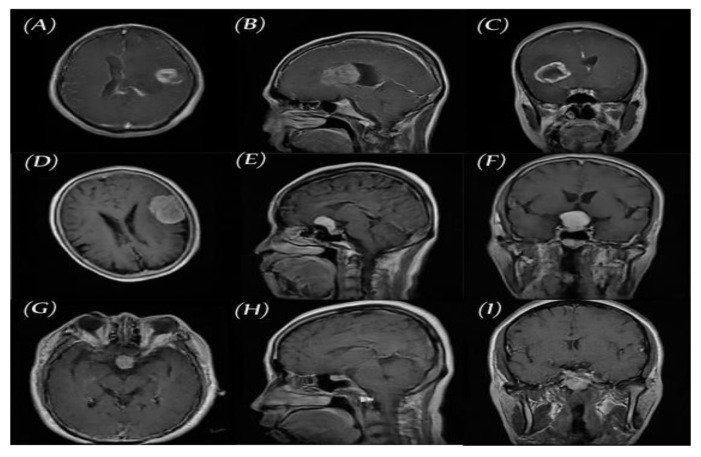
Samples of ‘realistic’ synthetic MR images, in three planes, produced by PGGAN: (**A**–**C**) glioma (**D**–**F**) meningioma, and (**G**–**I**) pituitary tumor.

**Figure 10:**
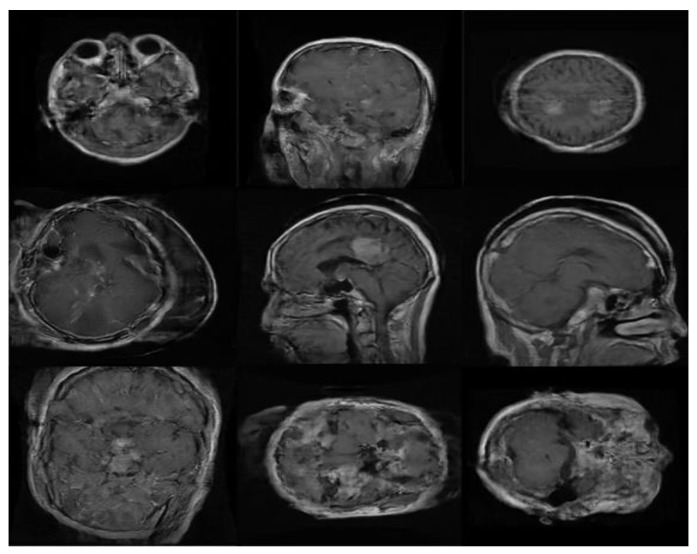
Samples of ‘wrong’ synthetic MR images produced by PGGAN.

Clinical validation of synthesized MRIs by an experienced radiologist: To ensure that the output of PGGAN mimicked realistic tumor MRIs and that correct features were generated, an experienced radiologist reviewed the images. The radiologist was asked to confirm that the three target tumor types were realistically represented through the generated images and to help identify poorly generated or ‘wrong’ images, which were then discarded. Figure 9 and Figure 10 illustrate samples of generated MR images (‘realistic’ and ‘wrong’ generated images, respectively). New ‘realistic’ MRIs of brain tumors generated using the PGGAN model were all added to the original training data set, then the three models were retrained and the performance of each model was checked.

**Figure 11:**
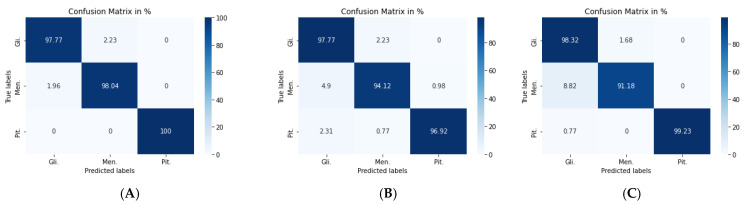
Confusion matrix for: (**A**) VGG19 + CNN model, (**B**) VGG19 + GRU model, and (**C**) VGG19 + Bi-GRU model.

With the VGG19 + CNN model, the Adam optimizer achieved the highest accuracy, which stood at 98.54%, which was 4.38% higher than that of classic DA. Additionally, overall performance metrics also rose in value, especially with respect to the pituitary tumors illustrated in Table 7 and Table 8. Figure 11a shows the confusion matrix of the VGG19 + CNN model. It is worth noting that, even with the best model, six images were misclassified. Four images of gliomas were erroneously classified as meningiomas and two meningiomas were wrongly classified as gliomas.

For the second model, the Nadam optimizer algorithm achieved the highest accuracy, which stood at 96.59%, as shown in Table 7. Performance metrics are detailed in Table 8. Figure 11b shows the related confusion matrix with a 97.77% accuracy rate for gliomas, 94.12% for meningiomas, and 96.92% for pituitary tumors.

For the third model, the overall performance metrics improved, as illustrated in Table 7 and Table 8. The model’s accuracy rose to 96.84%, with a 1.22% accuracy improvement over classical DA. Figure 11c shows the confusion matrix for the VGG19 + Bi-GRU model.

**Figure 12:**
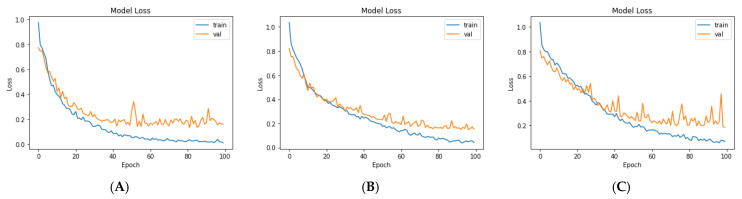
The losses of the three proposed models during training for: (**A**) VGG19, (**B**) VGG19 + GRU, and (**C**)VGG19 + Bi-GRU).

**Figure 13:**
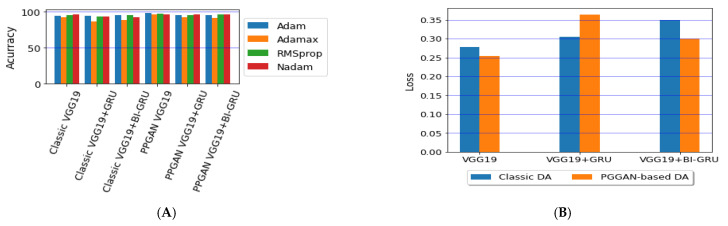
Comparison of the (**A**) accuracy and (**B**) average losses for the three proposed models.

Lastly, we compared the three proposed models in terms of accuracy and loss to determine the best of the three. Figure 12 shows the training and validation loss versus epochs for the three best-performing models. Figure 13 shows the accuracy rates of the three models, indicating that the VGG19 model had the highest performance accuracy and the lowest loss value of the three proposed models.

## 4. Discussion

This section compares the performances of our framework with its three proposed models (VGG19 + CNN, VGG19 + GRU, and VGG19 + Bi-GRU) with eight recent state-of-the-art models [4,23,30,31,32,34,38,39]. Based on the experimental results reported in these papers, we found that our proposed combined structure of VGG19 + CNN with the PGGAN data augmentation model was superior to all recent works that tackled brain tumor MRI classification.

We first compared the results of studies of four state-of-the-art models that used classical augmentation [4,23,31,38] with the results of our best comparable model: VGG19 + CNN) combined with classic augmentation. The authors of [31] developed a CNN model for the classification of MRIs of a number of brain tumors. Only axial 989 MRIs of brain tumors were used. Three types of operations, rotating, shifting, and mirroring, were used for the purposes of data augmentation in order to increase the data set. The authors applied three classifier models: a convolutional neural network (CNN), a fully connected network, and a random forest. The CNN yielded the best classifier when applied to 256 × 256 images with an accuracy of 91.43%. A genetic algorithm (GA) was used to choose the best CNN architecture to classify the different grades of gliomas [38]. The CNN was comprised of 12 layers, increasing accuracy to 94.2%. A new classifier based on the GAN model was introduced by [4]. A deep convolutional model was pre-trained as a discriminator within the DCGAN model. The discriminator was designed to distinguish between actual and generated images and define image features, and it was then fine-tuned as the classifier of brain tumors. This model achieved a 95.6% accuracy. A CNN-based deep learning model was developed by [23] in order to classify MRIs of tumors. Images of 128 × 128 pixels were fed into a 16-layer model. The investigated DL model achieved an accuracy of 96.13%.

As shown in Table 9, our VGG19 + CNN model with classic augmentation multiplied the data set images by nearly 6-fold and achieved a 96.59% accuracy, higher than the four models with which it was compared. This indicated the superiority of our framework even when using classical augmentation. Moreover, using the additional images generated by the PGGAN model helped to increase the accuracy of the VGG19 + CNN model by a further 1.96%.

As shown in Table 10, we compared the accuracy of our VGG19 + CNN model with all seven recent models that were designed to classify MRIs of brain tumors into three types (gliomas, meningiomas, and pituitary tumors). The accuracy rate of our combined VGG19 + CNN and PGGAN data augmentation framework surpassed that of the seven other models, achieving an accuracy of 98.54%. To further assess the performance of our framework in comparison with the seven other models, we compared all remaining metrics (precision, sensitivity, and specificity). We calculated the performance metrics (precision, sensitivity, and specificity) calculated with our VGG19 + CNN model and PGGAN data augmentation framework. Metrics pertaining to the identification of meningiomas stood at 96.15% for precision, 98.04% for sensitivity, and 98.71% for specificity. Glioma-related metrics stood at 98.87, 97.77, and 99.14% for precision, sensitivity, and specificity, respectively. Pituitary tumor-related metrics stood at a perfect 100%.

We lastly compared our VGG19 + CNN and PGGAN model with previous models that used the same Cheng et al. [30] data set. Results are shown in Table 10, including a comparison of accuracy and most of the performance metrics that were assessed in the proposed framework based on the VGG19 + CNN model and PGGAN augmentation. We achieved high performance with the VGG19 + CNN model and PGGAN augmentation without the use of manual segmentation. Our studies reported the use of manual segmentation of tumor regions to improve the performance of their models [30,34]. Ref. [32] reported inferior results for their KE-CNN model, compared with our framework. Importantly, a model in which the CNN was designed using a genetic algorithm [38] still yielded inferior results in comparison to those of our framework. We also compared our framework to a model based on DCGAN [4], where the PGGAN model could reportedly generate high-resolution images that helped to increase accuracy [23]. A classical augmentation was used in scenario I to improve performance, In scenario II, PGGAN was used for augmentation and the results showed that PGGAN augmentation plus the VGG19 + CNN model outperformed the other proposed models in our work. Ref. [39] used GoogLeNet + KNN to classify brain tumors. Prior to our study, they reported the highest accuracy in the literature. Our proposed VGG19 + CNN and PGGAN augmentation framework outperformed the [39] model.

Finally, using a PGGAN model to generate high-resolution images with ‘realistic’ features helped us overcome the problem of overfitting with better images than those produced by classical augmentation, resulting in higher levels of accuracy.

## 5. Conclusions

The recent research work in tumor classification based on MR images has witnessed some challenges, such as the number of images in the data set, and the low accuracy of the designed models. This work proposed a complete framework based on a deep learning model as feature extractors with different classifier models designed to classify MRIs of gliomas, meningiomas, and pituitary tumors using different types of augmentations. The feature extractor VGG19 was used to extract features of brain tumor MRIs. Three types of classifiers were then tested (CNN, and the recursive neural networks GRU and Bi-GRU). In our work, the number of images in the data set was increased by using different methods for image augmentation: PGGAN and classic augmentation methods such as rotation, mirror, and flipping. The proposed models achieved more accuracy than the recent introduced models.

The CNN classifier yielded the best accuracy performance (98.54%). The VGG19 + CNN model and PGGAN augmentation framework outperformed the other models in all previous work, with accuracy values of 98.54, 98.54, and 100% for gliomas, meningiomas, and pituitary tumors, respectively.

Our study tested the classification of only three general brain tumor types: meningiomas, gliomas, and pituitary tumors. This constitutes a limitation of the study since other types of brain tumors exist. Moreover, our synthetic images were 256 × 256, while the primary data set images were all 512 × 512. Because of limitations of computational resources, the input for the model was resized to 224 × 224. In future studies, we aim to work on acquiring primary images with appropriate size in order to make the images as realistic as possible to a radiologist.

## Figures and Tables

**Table 1:** Summarization of the previous models.

Ref.	Accuracy %	No. of Images
[30]	91.28	3064
[31]	91.43	989 *
[32]	93.68	3064
[33]	92.13	989 *
[34]	91.13	3064
[35]	86.56	3064
[36]	90.89	3064
[37]	84.19	2100
[38]	94.2	989 *
[23]	96.13	3064
[39]	98	3064
[4]	95.6	3064

* Only the axial plane is used.

**Table 2:** Architecture details of the VGG19+CNN model. The maxpooling layer is applied after each convolution layer.

VGG19 + CNN Model	Output Shapes
[Conv (3 × 3) − 64] × 2	224 × 224 × 64
[Conv (3 × 3) − 128] × 2	112 × 112 × 128
[Conv (3 × 3) − 256] × 4	56 × 56 × 256
[Conv (3 × 3) − 512] × 4	28 × 28 × 512
[Conv (3 × 3) − 512] × 4	14 × 14 × 512
Max pool	7 × 7 × 512
Flatten	25,088
Dense (relu)	4096
Dropout (0.5)	4096
Dense (relu)	4096
Dropout (0.5)	4096
Dense (SoftMax)	3

**Table 3:** Architecture details of the VGG19 + GRU model.

VGG19 + GRU Model	Output Shapes
[Conv (3 × 3) − 64] × 2	224 × 224 × 64
[Conv (3 × 3) − 128] × 2	112 × 112 × 128
[Conv (3 × 3) − 256] × 4	56 × 56 × 256
[Conv (3 × 3) − 512] × 4	28 × 28 × 512
[Conv (3 × 3) − 512] × 4	14 × 14 × 512
Max pool	7 × 7 × 512
Reshape	7 × 7 × 512
Time Distributed	7 × 3584
GRU (512)	512
Dense (relu)	1024
Dropout (0.5)	1024
Dense (relu)	1024
Dropout (0.5)	1024
Dense (SoftMax)	3

**Table 4:** Architecture details of the VGG19 + Bi-GRU model.

VGG19 + Bi-GRU Model	Output Shapes
[Conv (3 × 3) − 64] × 2	224 × 224 × 64
[Conv (3 × 3) − 128] × 2	112 × 112 × 128
[Conv (3 × 3) − 256] × 4	56 × 56 × 256
[Conv (3 × 3) − 512] × 4	28 × 28 × 512
[Conv (3 × 3) − 512] × 4	14 × 14 × 512
Max pool	7 × 7 × 512
Reshape	7 × 7 × 512
Time Distributed	7 × 3584
Bidirectional	1024
Dense (relu)	1024
Dropout (0.5)	1024
Dense (relu)	1024
Dropout (0.5)	1024
Dense (SoftMax)	3

**Table 5:** Comparison between different optimizers for the three proposed models.

Models	Adam	Adamax	RMSprop	Nadam
**VGG19 + CNN**	94.16	92.94	95.59	**96.59**
**VGG19 + GRU**	**94.89**	87.10	93.19	93.67
**VGG19 + BI-GRU**	95.38	89.05	**95.62**	92.94

The bold values indicate the best accuracy value that was achieved.

**Table 6:** All performance results for the three proposed models (where Men, Gli, and Pit refer to meningioma, glioma, and pituitary tumor, respectively).

Model	Class	Accuracy	Precision	Sensitivity	F1-Score	Specificity	NPV	MCC
**VGG19 + CNN**	Gli.	96.97	93.81	100	96.81	94.40	100	94.10
	Men.	97.44	98.92	90.2	94.36	99.70	97.02	92.87
	Pit.	99.06	100	96.92	98.44	100	98.68	97.80
**VGG19 + GRU**	Gli.	95.01	95.87	89.92	92.8	97.84	94.58	89.01
	Men.	94.46	87.27	94.12	90.1	94.59	97.61	86.78
	Pit.	98.89	98.46	98.46	98.46	99.13	99.13	97.60
**VGG19 + Bi-GRU**	Gli.	96.11	94.05	97.21	95.6	95.26	97.79	92.15
	Men.	96.59	95.83	90.20	92.93	98.71	96.83	90.76
	Pit.	98.54	97.69	97.69	97.69	98.93	98.93	96.62

**Table 7:** Comparison between different optimizers for the three proposed models using PGGAN DA.

Models	Adam	Adamax	RMSprop	Nadam
**VGG19 + CNN**	**98.54**	97.57	97.57	96.59
**VGG19 + GRU**	95.62	92.21	95.13	**96.59**
**VGG19 + BI-GRU**	95.62	91.97	**96.84**	96.35

The bold values indicate the best accuracy value that was achieved.

**Table 8:** All performance results for the three proposed models using PGGAN DA.

Model	Class	Accuracy	Precision	Sensitivity	F1-Score	Specificity	NPV	MCC
**VGG19 + CNN**	Gli.	98.54	98.87	97.77	98.31	99.14	98.29	97.03
	Men.	98.54	96.15	98.04	97.09	98.71	99.35	96.12
	Pit.	100	100	100	100	100	100	100
**VGG19 + GRU**	Gli.	97.08	95.63	97.77	96.69	96.55	98.25	94.1
	Men.	97.23	95.05	94.12	94.58	98.38	98.06	92.81
	Pit.	98.78	99.21	96.92	98.05	99.64	98.59	97.18
**VGG19 + Bi-GRU**	Gli.	96.84	94.62	98.32	96.44	95.69	98.67	93.65
	Men.	97.08	96.88	91.18	93.94	99.03	97.14	92.09
	Pit.	99.76	100	99.23	99.61	100	99.64	99.44

**Table 9:** Comparison of our framework with other works based on classic augmentation.

Ref.	Augmentation Operation	Data Set Size after Augmentation	Data Set Division	ACC%
[31]	Rotation random angle between (0 and 360 angle). Shifted randomly by −4 to 4 pixels left or right and up or down. Mirror across its *y*-axis.	Not mentioned	Use images for 191 patients and divided them according to patients as follows: training: 149 patients, validation: 21 patients, and testing: 21 patients.	91.43
[38]	Rotation 10, 20, or 30 clockwise or counterclockwise. Mirroring, and translating 15 pixels to right or left. Scaling to 0.75 of the original.	Augmentation is done after the train and test images are randomly selected. For each tumor class take: 1521 as training images, and 115 as testing images	Divide the data set images into: training group, and testing group.	94.2
[23]	Rotation image with angle 45. Mirroring right/left. Flipping up–down. Adding salt noise.	Images are shuffled, splitting, and finally, the author applies augmentation. The authors increased the original number to 15,320 images.	Divide the data set images into training group: 68%, validation group: 16%, and testing group: 16%.	96.13
[4]	Rotation random angle between (0 and 359 angle). Mirror 25% on each axis.	Not mentioned	Not mentioned	95.6
Our framework (VGG19 + CNN and classic DA)	Rotation 90, 180, 270 angles. Mirroring right/left. Flipping up–down.	Images are randomly selected, then split, and finally, we augment the only training and validation groups. The original images increased to 19,215 images for the training group, and 4536 for the validation group.	Divide the data set images into: training group: 70%, validation group: 15%, and testing group: 15%.	96.59
Our framework (VGG19 + CNN and PGGAN DA)	Rotation 90, 180, 270 angles. Mirroring right/left. Flipping up–down. Add generated images from PGGAN models (8100 images).	We increased the original images to 27,315 images for training, and 4536 images for validation.	Divide data set into: training: 70% + PGGAN-generated images, validation: 15%, and testing: 15%.	98.54

**Table 10:** Comparison of our framework with other works by precision, sensitivity, and specificity.

Method	PPV			Sensitivity			SPC			ACC%
	Gli.	Men.	Pit.	Gli.	Men.	Pit.	Gli.	Men.	Pit.	
[30]	-	-	-	96.4	86.0	87.3	96.3	95.5	95.3	91.28
[32]	91.0	94.5	98.3	97.5	76.8	100	-	-	-	93.68
[34]	-	-	-	95.1	86.97	91.24	96.29	96.0	95.66	91.13
[38]	91.9	95.3	95.7	98.3	87.8	96.5	95.7	97.8	97.8	94.2
[23]	97.2	95.8	95.2	94.4	95.5	93.4	95.1	98.7	97.0	96.13
[39]	99.2	94.7	98.0	97.9	96.0	98.9	99.4	98.4	99.1	98.0
[4]	95.89	92.43	95.29	96.83	89.98	97.93	96.38	97.79	97.54	95.6
Our framework	98.87	96.15	100	97.77	98.04	100	99.14	98.71	100	98.54

## Data Availability

Data available in a publicly accessible repository. The data presented in this study are available in FigShare at [http://dx.doi.org/10.6084/m9.figshare.1512427].
